# Preliminary results from digestive adaptation: a new surgical proposal for treating obesity, based on physiology and evolution

**DOI:** 10.1590/S1516-31802006000400004

**Published:** 2006-05-04

**Authors:** Sérgio Santoro, Manoel Carlos Prieto Velhote, Carlos Eduardo Malzoni, Fábio Quirino Milleo, Sidney Klajner, Fábio Guilherme Campos

**Keywords:** Morbid obesity, Surgery, Ghrelin, Glucagon-like peptide 1, Peptide YY, Obesidade mórbida, Cirurgia, Peptídeo YY, Citocinas, Fisiologia

## Abstract

**CONTEXT AND OBJECTIVE:**

Most bariatric surgical techniques include essentially non-physiological features like narrowing anastomoses or bands, or digestive segment exclusion, especially the duodenum. This potentially causes symptoms or complications. The aim here was to report on the preliminary results from a new surgical technique for treating morbid obesity that takes a physiological and evolutionary approach.

**DESIGN AND SETTING:**

Case series description, in Hospital Israelita Albert Einstein and Hospital da Polícia Militar, São Paulo, and Hospital Vicentino, Ponta Grossa, Paraná.

**METHODS:**

The technique included vertical (sleeve) gastrectomy, omentectomy and enterectomy that retained three meters of small bowel (initial jejunum and most of the ileum), i.e. the lower limit for normal adults. The operations on 100 patients are described.

**RESULTS:**

The mean follow-up was nine months (range: one to 29 months). The mean reductions in body mass index were 4.3, 6.1, 8.1, 10.1 and 10.7 kg/m^2^, respectively at 1, 2, 4, 6 and 12 months. All patients reported early satiety. There was major improvement in comorbidities, especially diabetes. Operative complications occurred in 7% of patients, all of them resolved without sequelae. There was no mortality.

**CONCLUSIONS:**

This procedure creates a proportionally reduced gastrointestinal tract, leaving its basic functions unharmed and producing adaptation of the gastric chamber size to hypercaloric diet. It removes the sources of ghrelin, plasminogen activator inhibitor-1 (PAI-1) and resistin production and leads more nutrients to the distal bowel, with desirable metabolic consequences. Patients do not need nutritional support or drug medication. The procedure is straightforward and safe.

## RESUMO

**CONTEXTO E OBJETIVO:**

A maioria das técnicas de cirurgia bariátrica inclui anastomoses ou bandas ou exclusões de segmentos digestivos, principalmente o duodeno, que podem gerar sintomas ou complicações. O objetivo do estudo foi apresentar os resultados dos dois primeiros anos de uso de uma nova técnica no tratamento cirúrgico da obesidade: gastrectomia vertical com omentectomia e enterectomia segmentar.

**TIPO DE ESTUDO E LOCAL:**

Descrição de série de casos operados no Hospital Israelita Albert Einstein, Hospital da Polícia Militar, São Paulo, e Hospital Vicentino, Ponta Grossa, Paraná, Brazil.

**MÉTODO:**

A técnica utiliza a gastrectomia vertical, omentectomia maior e enterectomia segmentar (incluindo jejuno basicamente) que deixa três metros de intestino delgado (jejuno inicial e a maior parte do íleo), em comprimento no limite inferior do que se considera normal para humanos adultos. Os 100 primeiros pacientes são descritos.

**RESULTADOS:**

Com acompanhamento de até 29 meses pós-operatórios (média de oito meses) a redução no índice de massa corpórea foi de 4.3, 6.1, 8.1, 10.1, 10.7 kg/m^2^, respectivamente nos meses 1, 2, 4, 6 e 12 de pós-operatório. Todos os pacientes acusam saciedade precoce. Houve melhora acentuada das doenças associadas em especial a diabetes. Complicações cirúrgicas ocorreram em 7% dos pacientes todas resolvidas sem seqüelas. Não houve óbitos.

**CONCLUSÕES:**

O procedimento cria um trato gastrointestinal proporcionalmente reduzido, mas com suas funções digestivas intactas. O procedimento retira fontes de produção de grelina, inibidor do ativador do plasminogênio-1 (PAI-1), resistina e permite que mais nutrientes sejam absorvidos no intestino distal, com as desejáveis conseqüências metabólicas. Os pacientes não precisam de suporte nutricional ou de medicações. O procedimento é fácil e seguro.

## INTRODUCTION

Although there has not been any significant documented change in human physiology over the last hundred years, fast environmental changes have somehow made some of our physiological tools unfit for the new circumstances. Among other pathological conditions, obesity and its associated diseases underwent a great increase in their incidence over the course of the twentieth century. Nutritional education and treatment by means of medications have many times failed, and this has led to the appearance of many surgical techniques for the treatment of extreme obesity. However, none of them are in essence physiologically based.

Current surgical treatments create “another disease” to counterbalance obesity. Some treatments provoke nonspecific malabsorption, thereby leading to loss of non-caloric nutrients like calcium, iron, folic acid, etc., as well as the diarrhea^1^ and flatulence they create. Some procedures place obstacles against food ingestion, through the use of narrowing anastomoses or sometimes the use of prostheses.^2^ These imply dysphagia, vomiting, stasis esophagitis, etc. Many of the current procedures involve digestive tract exclusions that cause atrophy of the mucosa, with bacterial proliferation that leads to intense flatulence and sometimes bacterial translocation to the portal system. The latter has been linked to hepatic decompensation.^3,4^ Furthermore, exclusion impedes endoscopy.

Physiologically-designed surgical alternatives are needed. They should be easy to perform, should not create nutrient exclusions in any segment, and should avoid the creation of endoscopic blind areas. Ideally, they should also avoid strangling prostheses and narrowing anastomoses that present mechanical obstacles to the ingestion of food. They should avoid malabsorption and, fundamentally, they should not cause harm to important digestive functions unrelated to obesity, like the gastric, pyloric and duodenal functions. Instead, an ideal procedure should solely provide positive interference with the neuroendocrine control of hunger and satiety and only affect patients’ lives with regard to these issues. If such a procedure existed, it could perhaps be used at an earlier stage, for less obese patients, under safer conditions and before obesity leads to severe damage.

### Physiological background to the new proposal

**GLP-1**: Glucagon-like peptide 1 (GLP-1) is a polypeptide hormone secreted mainly by the distal gut in response to nutrient ingestion. It has six fundamental actions: it is insulinotropic^5^ and glucogenostatic,^6,7^ it reduces gastric acid output,^8^ it causes major reduction in gastric emptying,^9^ it causes relaxation of the gastric fundus (allowing the stomach to receive a larger volume without increasing the sensation of distension)^10^ and, lastly, GLP-1 crosses the blood-brain barrier and causes satiety.^11^

The evolutionary aspects of GLP-1 functions are extraordinary. Because of sporadic access to food in nature, primitive man had the instinct to eat as much as possible to create reserves for times of hunger. While he was hungry, the digestive transit had to be fast, in order to create space for further eating, but when nutrients reached the distal gut, it was time to slow down the transit; otherwise, nutrients could be lost in stools. Enterohormones produced by the distal gut do this job by slowing gastric emptying, provoking intense insulin secretion and blocking the action of glucagon to help the organism to stock up absorbed nutrients. Furthermore, GLP-1 relaxes the gastric fundus to allow it to hold the food that cannot be processed right away, causes central satiety to stop the eating process and diminishes gastric acid output, since the meal is about to be finished.

**Ghrelin**: This is a 28-amino acid peptide, predominantly produced by the stomach, that displays strong growth hormone-releasing activity. It also stimulates gastric acid secretion and is able to induce adiposity by activating a central mechanism for increasing food intake and decreasing fat utilization.^12–14^ After a meal, ghrelin production falls. As time passes after the last meal, its production is enhanced, and it has been shown that this contributes towards the genesis of hunger.^15^ High levels of ghrelin are not a common cause for obesity since it has been shown that obese people have low levels of this hormone. However, when significant weight loss occurs, ghrelin levels become high, thus generating hunger and energy savings. Ghrelin is probably an obstacle to weight loss and subsequent weight maintenance.^16^

**PAI-1**: Plasminogen activator inhibitor 1 (PAI-1) is the primary physiological inhibitor of plasminogen activation, which means it is a pro-coagulant factor. Circulating PAI-1 levels are elevated in patients with coronary heart disease and it plays an important role in the development of atherothrombosis by decreasing fibrin degradation.^17^ PAI-1 is produced mainly by visceral fat tissue, chiefly the omentum and mesenteric fat.^17–19^ Procedures that cause reduction in PAI-1 levels have already been put forward for improving the metabolic profile and reducing the cardiovascular risk.^20,21^

**Resistin**: This is a hormone produced by adipose cells. It acts on skeletal muscle myocytes, hepatocytes and adipocytes themselves, to reduce their sensitivity to insulin, and it has been linked to diabetes.^22^ Abdominal fat is the main source of resistin.^23^

**Visceral obesity**: The fat tissue in the abdomen is clearly associated with what is called plurimetabolic syndrome. The waist-to-hip ratio has been used to quantify cardiovascular risk and many epidemiological studies have indicated that it has a relationship with high blood pressure, hypertriglyceridemia, insulin resistance and atherothrombotic disease. Insulin action inhibits lipolysis and, since visceral fat is insulin-resistant, it keeps on releasing free fatty acids (FFA) to the portal system. It is believed that the insulin resistance of the liver derives from a relative increase in the delivery of FFA from the omental fat depot to the liver (via the portal vein).^24^ Many extremely obese patients have quite a good metabolic profile because they mostly have subcutaneous fat. On the other hand, it is also clear that, except for orthopedic, respiratory and reflux complications, most metabolic complications of obesity are related to visceral fat.

**Peptide YY (PYY) and oxyntomodulin**: These two enterohormones are also produced mainly when nutrients reach the distal bowel. Both induce satiety,^25,26^ and PYY probably enhances the insulin response.^27^

### Evolutionary background to the new proposal

The primitive diet was raw, full of poorly digestible fiber and very hypocaloric. The ingested volumes had to be large. Consequently, the gastrointestinal tract had to be voluminous and long to accommodate and process voluminous food and to efficiently extract nutrients from cellulose-encased vegetal cells. Herbivores, for these reasons, have longer bowels than carnivores.^28^ Among primates, including humans, those who eat lower quality food, have longer gastrointestinal tracts.^29,30^ Analysis of our ancestors shows that, as the quality of diet became enhanced, simultaneously with the development of larger brains, there was also a diminution in gastrointestinal tracts.^31,32^

Over a few recent decades, the human diet has become refined and enriched and has changed faster than ever.

The modern diet has become unnaturally hypercaloric, poor in fiber and extremely easy to absorb. After a meal with these characteristics, absorption takes place quickly in the proximal portions of the bowel, creating peaks of nutrient absorption. The distal bowel tends to have minimal absorption work, which may cause a lack of production of GLP-1 and PYY. Indeed, it has been noticed that diabetic and obese individuals have lower postprandial production of GLP-1.^33,34^ Following the evolutionary line, maybe it would be better to have a further proportional reduction in the gastrointestinal tract.

Under these new circumstances, the modern human small bowel has become excessively long. It has been shown that obese people tend to have longer small bowels than thin people, and bowel length is related to weight and not to height.^35^ This fact probably worsens the picture and contributes towards a situation in which even smaller amounts of nutrients reach the distal bowel. This in turn implies that there is less signaling of satiety, through lower production of GLP-1 and PYY.

Nature is doing what it does best: selection. Individuals with strong eating instincts are being killed by lack of adaptation of their digesti ve system and their neuroendocrine control of hunger, satiety and energy stores. Also, as this lack of adaptation is obvious and related to lesser chances of survival, obesity has reduced sexual attraction in modern times.

### A new surgical proposal for treating obesity

Surgery is capable of adapting the digestive system and the individual's instincts to eat in abundance and to prefer hypercaloric food, without causing damage to precious digestive functions, like the gastric, pyloric, duodenal, ileal and colonic functions. These many digestive functions are precious, even for obese individuals. However, we believe that gastric capacity is larger than necessary^36^ and that the intestines are too long and too permeable for modern diets.^37^ In 2003, we described and published^38^ the early results from a surgical proposal for treating obesity that included vertical gastric resection (in the same way as already extensively used in the “duodenal switch” technique^39^) in association with omentectomy and midgut enterectomy in three patients. This procedure leaves three meters of small bowel, which is the lower limit for the length of the normal small bowel. We now report on the preliminary results of a larger sample of patients using this new technique.

## PATIENTS AND METHODS

The Research Ethics Committees of Hospital da Polícia Militar and Hospital Israelita Albert Einstein, both in São Paulo, Brazil, and also of Hospital Vicentino, Ponta Grossa, Paraná, Brazil, approved the protocol. A detailed informed consent declaration was signed by the patients.

**Patients:** One hundred patients were operated on from October, 2002, to March, 2005. There were 52 women (52%) and 48 men (48%), with ages ranging from 22 to 66 years (mean: 43.7 years).

At the time of surgical treatment, the patients presented ranges of 80 to 184 kg (mean: 114.2 kg) in body weight, 146 to 192 cm in height (mean: 168 cm) and 35 to 51 kg/m^2^ in body mass index (mean: 40.1 kg/m^2^). The comorbidities diagnosed included orthopedic problems in 32 patients (32%), essential hypertension in 43 (43%), diabetes in 34 (34%), hypertriglyceridemia in 59 (59%), hypercholesterolemia in 49 (49%) and respiratory problems in 21 (21%).

**Technique:** The procedure began through laparoscopic access. Five trocars were positioned: two 12-mm trocars (one in the mid-line, 8 cm above the umbilicus, and the other in the upper left quadrant) and three 5-mm trocars (one in the upper right quadrant, one in the epigastrium for the liver retractor and one lateral in the upper left quadrant).

**Figure 1:**
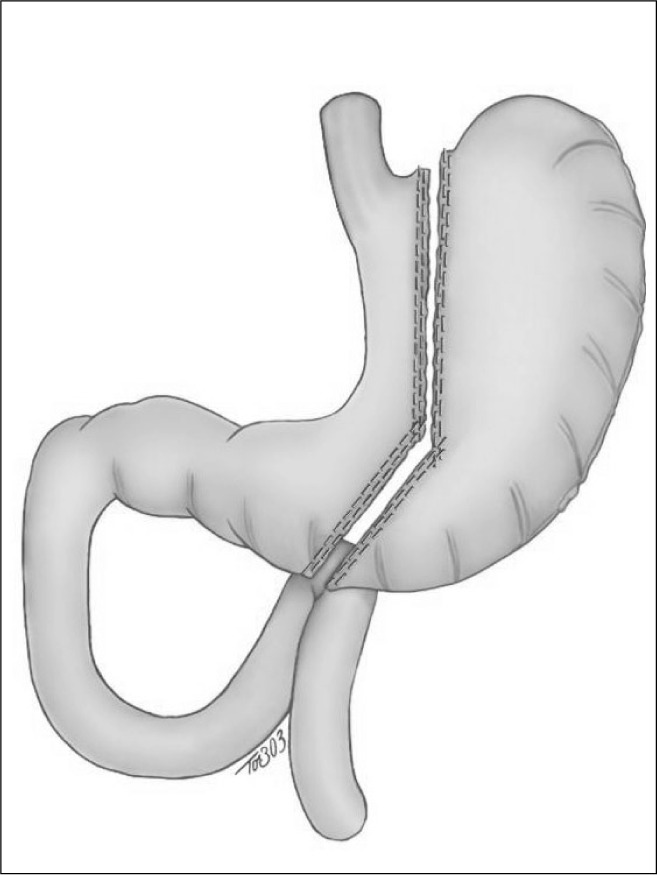
Sleeve gastrectomy being performed using a laparoscopic linear cutting stapler.

First, the omental bursa was opened and the greater omentum was sectioned with the help of a sealer and divider tool (Ultracision^®^ or Ligasure^®^). Dissection started just beside the gastric greater curvature at a point located 6 cm from the pylorus and went up to the angle of His. A sleeve gastrectomy was performed using a laparoscopic linear cutting stapler (Figure 1). A Fouchet tube was passed into the stomach to ensure that the gastric tube left in the lesser curvature was approximately 3 to 4 cm wide. A Penrose drain (brought to the exterior at the site of the left-flank 12-mm trocar) and a nasogastric tube were positioned under laparoscopic view.

**Figure 2:**
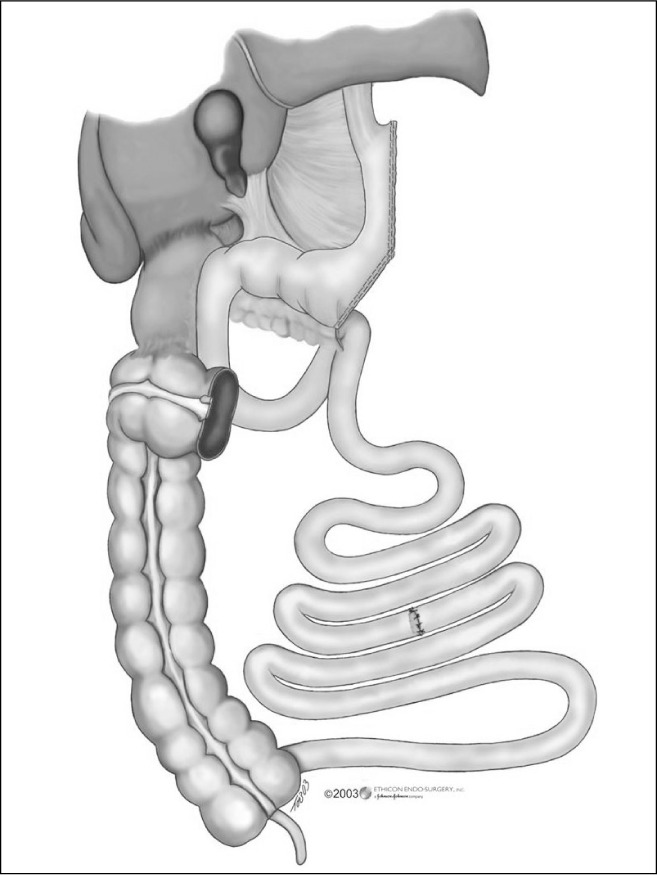
Final appearance of the proportionally reduced gastrointestinal tract by the proposed surgical technique.

Following this, a small midline laparotomy (6 to 8 cm), immediately above the umbilicus, was made by downwards extension of the 12-mm incision that had been made in the midline for one of the trocars. This was done in order to remove the gastric specimen and the greater omentum (after detaching it from the colon), and to perform an enterectomy, leaving the first 50 to 100 cm of the jejunum and the last 200 to 250 cm of the ileum. In the first cases, we retained 150 cm of each segment.^38^ Subsequently, we started to retain the following proportions: 50/250 cm (jejunum/ileum) in diabetic or hypertriglyceridemic patients and 100/200 cm in the others, with the aim of retaining a total of three meters (Figure 2).

Antibiotic and deep vein thrombosis prophylaxis was used in all patients before surgery: cephalothin 1 g every 6 hours for one day; and enoxaparin 40 mg once daily, preoperatively and for one week after the procedure. End-toend enteroenteric anastomosis was performed. The mesenteric borders were closed to avoid internal hernias. The abdominal wall and laparoscopic incisions were then closed.

During the first 24 hours after the operation, all patients received cefazolin as a prophylactic antibiotic and received only intravenous fluids over the first 48 hours. Following this, they were all advised just to take liquids, in quantities of not more than 40 ml every 20 minutes, for a week. Thereafter, they were allowed to eat solids. The recommendation given was that they should make small meals starting with a portion of salad. Also, portions of fruits, vegetables, fish and chicken were recommended. We suggested that whole-wheat flour should be consumed, instead of refined wheat flour and, finally, we ask them to avoid simple (refined) sugar.

## RESULTS

The duration of the operative procedures ranged from 150 to 300 minutes (mean: 210 minutes). The physical features of the resected specimens are presented in Table 1. Postoperative complications occurred in seven patients (7 %) and are listed in Table 2. Four patients developed gallbladder lithiasis and one required surgical resection 14 months later. There was no mortality.

**Table 1.** Physical features of the resected specimens in the reported cases

Structure	Length (mean)	Weight (mean)	Volume (mean)
Stomach	-	75 to 220 grams (140)	1.1 to 2.3 liters (1.5)
Small bowel	120 to 500 cm (270)	350 to 1300 grams (810)	-
Omentum	-	80 to 1280 grams (600)	-

**Table 2.** Postoperative complications in this series

Complications	n (%)	Management
Perisplenic abscess (*Staphylococcus aureus*)	1 (1%)	Surgical drainage on the 26^th^ POD
8-mm gastric laceration outside the mechanical suture	1 (1%)	Suture and drainage on the 1^st^ POD
Abdominal wall bleeding at trocar site	1 (1%)	External compression with resolution
Internal bleeding	2 (2%)	Reoperation on 1^st^ and 2^nd^ POD
Supraumbilical hernia	1 (1%)	Surgical repair during dermolipectomy in the 7^th^ month
Gastric fistula	1 (1%)	Surgical drainage and enteral feeding

*POD = postoperative day.*

**Weight loss evolution**: All patients were followed for at least one month, with a mean body mass index (BMI) evolution from 39.9 kg/m^2^ to 35.6 kg/m^2^ over this first month (Δ = - 4.3 kg/m^2^). Ninety-seven patients were followed for at least two months and their mean BMI evolution was from 39.8 kg/m^2^ to 33.7 kg/m^2^ over the first two months (Δ = - 6.1 kg/m^2^). Eighty-one patients were followed for at least four months and their mean BMI evolution was from 40.2 kg/m^2^ to 32.1 kg/m^2^ over the first four months (Δ = - 8.1 kg/m^2^). Sixty-four patients were followed for at least six months and their mean BMI evolution was from 41.3 kg/m^2^ to 31.2 kg/m^2^ over the first six months (Δ = - 10.2 kg/m^2^). Thirty-nine patients were followed for at least 12 months (the mean follow-up for this group was 20.1 months) and their mean BMI evolution was from 41.0 kg/m^2^ to 30.3 kg/m^2^ (Δ = - 10.7 kg/m^2^).

The length of follow-up ranged from 1 to 29 months (mean: nine months). During this period, the comorbidities detected before surgical treatment showed clinical resolution or improvement. Table 3 shows how much improvement was observed in the comorbidities. Resolution was defined as the disappearance of the problem or the withdrawal of medication. Improvement was defined as a reduction in medication or better objective laboratory results or fewer symptoms.

**Table 3.** Clinical resolution and improvement of comorbidities after the proposed surgical treatment

Condition	Before surgery		After surgery			
			resolved		improved	
	n	%	n	%	n	%
Orthopedic problems	32	32%	23	71.9%	9	28.1%
Essential hypertension	43	43%	32	74.4%	11	25.6%
Diabetes	34	34%	31	91.2%	3	8.8%
Hypertriglyceridemia	59	59%	48	81.3%	11	18.7%
Hypercholesterolemia	49	49%	28	57,1 %	21	42.9%
Respiratory problems	21	21%	19	90.5%	2	9.5%

After surgery, six patients presented minimal complaints such as moderate intestinal constipation, and one patient had occasional dyspepsia that did not require continuous medication. No postoperative diarrhea was observed. Generally, all the patients reported great reduction in total daily ingestion and early and prolonged satiety. All of them were pleased that they had undergone the procedure.

## DISCUSSION

The surgical procedure reported here may bring many advantages. We believe it provides an adaptation of the digestive tract to the modern diet. Since food now is a lot more caloric than in the primitive diet, the gastric capacity required can be significantly reduced. However, in this surgical procedure, there is no obstacle to food progression, no narrowing anastomosis and no prosthesis. The stomach is proportionally reduced, while keeping its general structure (cardia, body, antrum and pylorus). Innervation by the lesser curvature remains intact. Early satiety caused by gastric distension tends to occur. Between meals, a smaller elevation of ghrelin production is expected because its major source (the gastric fundus) has been removed.^14,15^

The enterectomy proposed does not have the goal of causing malabsorption, and in fact it would not cause this. There is no report of enteric insufficiency with a proportional bowel of length 300 cm (containing duodenum, jejunum, ileum, ileocecal valve and colon). In fact, some normal people only have three meters of small bowel.^40^ The human small bowel ranges in length from three to eight meters. Indeed, some authors even consider shorter bowel lengths still to be normal.^41^ As expected, up to the present date, none of our patients have developed undernutrition or diarrhea and, taking their intestinal adaptation into account, it is less likely that they will still develop it later on.

The rationale for this enterectomy was to create a proportionally smaller but still normal intestine that has a smaller capacity and makes it more likely that nutrients will reach the ileum. This will cause earlier and more effective secretion of GLP-1 and PYY, which reduces the rate of gastric emptying, improves insulin secretion and promotes central satiety.

When an enterectomy is performed, part of its mesentery comes out, which means that visceral fat is removed. Omentectomy promotes additional resection of visceral fat and reduction of a source of PAI-1,^18,19^ thereby reducing the risk of atherothrombosis. It provides reduction in the sourcing of free fatty acids to the portal vein and the sourcing of resistin. Both events are thought to reduce hyperinsulinism and insulin resistance.^19,23^ As the specimens removed are bulky, the intra-abdominal pressure (IAP) is reduced. High IAP is related to respiratory, hemodynamic and reflux problems.

The results obtained are encouraging, since the patients achieved good weight losses without mechanical obstacles to food ingestion or malabsorption. Besides the benefits from the weight loss itself, we observed major improvements in their diabetic, hypertriglyceridemic, hypercholesterolemic, arterial hypertension, orthopedic and respiratory problems. The expected reductions in ghrelin, resistin and PAI-1 secretion and the early elevation of GLP-1 and PYY are now under investigation by our group.

For super-obese patients, the method presented here may not enable them to lose the amount of weight needed, since it is neither malabsorptive nor restrictive enough. Indeed, we have moved the patients with incapacity to perform physical activity, and those with a previous diagnosis of slow gastric emptying, over to a different technique derived from the method presented here: digestive adaptation with in-transit intestinal reserve.^42^ Notwith-standing this, most of the traditional bariatric techniques may still be indicated after the procedure we have described here.

The current surgical procedures for treating obesity are aggressive to digestive functions like esophageal function (in cases of providing obstacles to food ingestion) and gastric, pyloric, duodenal, jejunal and hepatic function (in cases of providing nutrient exclusions). Therefore, they are not recommended for patients prior to attaining severe obesity. Thus, such procedures have been performed at a late stage. Frequently, these patients already present damaged arteries and joints, and have already suffered the consequences of diabetes, hypertension, dyslipidemia, reflux and other obesity-associated conditions, besides the psychosocial problems. All in all, the surgical risk is higher.

As the procedure proposed here is simple and safe, and as it maintains the general structure and important digestive functions unharmed, with no need for nutritional support, we believe it can be recommended for patients before reaching extreme obesity. This should reduce the surgical risks even further (such risks are already low due to the simplicity of the procedure itself).

Weight loss occurs not because the patient cannot eat what he wants or cannot absorb nutrients completely. Patients stop eating earlier for the same reasons that anyone does so: the feeling that the stomach is full, and a hypothalamic-generated sensation of satiety, caused by the perception of nutrients, especially in the distal bowel. These patients’ quality of life (QOL) seems to be much improved by the weight loss and its consequences. In parallel, QOL is not worsened by any symptoms or situations (such as dysphagia, vomiting, diarrhea, flatulence or the need for life-long follow-up or pills) that are sometimes created by traditional procedures. Almost all of these patients do not present any sign or any symptom, except for early satiety.

Indeed, this is the first surgical procedure that does not aim to cure a sick or deficient organ, but intends to adapt a system to new circumstances. It is also based on compared physiology, by copying movements from evolution. It is an evolutionary and adaptive surgery technique that would not have become necessary if human beings had kept their primitive hypocaloric and scanty diet. It might become a very useful procedure, since it may interrupt the development of obesity, hypertriglyceridemia, hypercholesterolemia, type II diabetes, hyper-tension, atherothrombotic disease and other typical conditions of modern life.

Detailed research into hormonal changes is needed because a great deal of the physiological data comes from animal experiments. More patients, longer follow-up and multicenter experience are also necessary, in order to establish this procedure as a possible evolutionary surgical intervention capable of adapting the human gastrointestinal tract to the modern diet.

## CONCLUSION

The surgical technique proposed in this article brought together three well-known procedures: vertical gastrectomy, omentectomy and enterectomy. In association, they produced a proportionally reduced digestive tract, but without changing its general structure. There were no obstacles to food ingestion, no prostheses, no excluded segments, no detected malabsorption and no endoscopically blind areas. No harm to important digestive functions was observed over short and medium-term follow-up. The patients who underwent the operation lost weight and their comorbidities were resolved or very much improved. They did not need nutritional support or chronic intake of medication because of the procedure. The procedure was simple and the complication rate was low. There was no mortality.
